# Auto-inflammatory diseases

**DOI:** 10.1186/1546-0096-12-S1-I24

**Published:** 2014-09-17

**Authors:** Michael Hofer

**Affiliations:** 1 Pediatric Rheumatology Romande, CHUV, University of Lausanne, Lausanne, Switzerland

## 

Auto-inflammatory diseases are a rapidly moving field, where the description of new entities, the understanding of the pathogenesis and the treatment has shown important progress in the past years. This group of conditions include monogenic auto-inflammatory diseases, like FMF, CAPS, TRAPS and MKD, and non-monogenic auto-inflammatory diseases, like PFAPA, SoJIA, CRMO, and Behçet. The most common clinical feature is recurrent fever and a pro-inflammatory cytokine, IL-1, is a key molecule involved in the pathogenesis of these diseases. The treatment aims to control chronic inflammation and blocking agents of IL-1 have shown a great efficacy in most of these diseases.

The aim of the presentation is to make an update on the diagnosis and the treatment and review the recent recommendations elaborated by experts’ consensus through the SHARE project.

## Disclosure of interest

None declared.

